# Entropy-Based Uncertainty Quantification in Linear Consecutive k-out-of-n:G Systems via Cumulative Residual Tsallis Entropy

**DOI:** 10.3390/e27101020

**Published:** 2025-09-28

**Authors:** Boshra Alarfaj, Mohamed Kayid, Mashael A. Alshehri

**Affiliations:** 1 Department of Statistics and Operations Research, College of Science, King Saud University, P.O. Box 2455, Riyadh 11451, Saudi Arabia; 2 Department of Quantitative Analysis, College of Business Administration, King Saud University, Riyadh 11362, Saudi Arabia; mealshehri@ksu.edu.sa

**Keywords:** information-theoretic reliability, entropy-based uncertainty quantification, cumulative residual Tsallis entropy, consecutive k-out-of-n:G systems, stochastic ordering, dispersive ordering test, reliability modeling, lifetime data analysis

## Abstract

Quantifying uncertainty in complex systems is a central problem in reliability analysis and engineering applications. In this work, we develop an information-theoretic framework for analyzing linear consecutive k-out-of-n:G systems using the cumulative residual Tsallis entropy (CRTE). A general analytical expression for CRTE is derived, and its behavior is investigated under various stochastic ordering relations, providing insight into the reliability of systems governed by continuous lifetime distributions. To address challenges in large-scale settings or with nonstandard lifetimes, we establish analytical bounds that serve as practical tools for uncertainty quantification and reliability assessment. Beyond theoretical contributions, we propose a nonparametric CRTE-based test for dispersive ordering, establish its asymptotic distribution, and confirm its statistical properties through extensive Monte Carlo simulations. The methodology is further illustrated with real lifetime data, highlighting the interpretability and effectiveness of CRTE as a probabilistic entropy measure for reliability modeling. The results demonstrate that CRTE provides a versatile and computationally feasible approach for bounding analysis, characterization, and inference in systems where uncertainty plays a critical role, aligning with current advances in entropy-based uncertainty quantification.

## 1. Introduction

Uncertainty quantification is a central theme in reliability engineering, where system performance often depends on the interplay between randomness in component lifetimes and the structural configuration of the system. Among the many system models studied in this context, consecutive k-out-of-n systems and their extensions have emerged as fundamental frameworks for capturing the reliability of complex engineered structures. These configurations are particularly relevant in applications where localized or sequential failures can critically undermine system functionality, for example, in telecommunication networks, oil and gas pipelines, particle accelerators, and large-scale computing architectures. While the probabilistic properties of such systems are well understood, their information-theoretic characterization remains limited. Entropy-based measures, especially those tailored to lifetime distributions, offer powerful tools for analyzing uncertainty and complexity beyond variance-based metrics. In this regard, the CRTE provides a flexible and robust measure for quantifying uncertainty, but its role in linear consecutive k-out-of-n:G systems has not yet been systematically explored.

**Figure 1:**
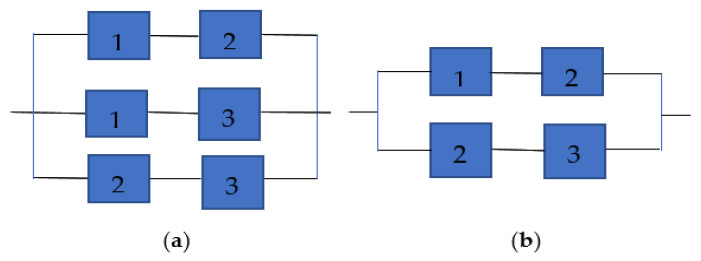
(**a**) Schematic of 2-out-of-3:G systems. (**b**) Schematic of linear 2-out-of-3:G systems.

In reliability engineering, consecutive k-out-of-n systems and their extensions have long been recognized as essential models for analyzing the reliability of complex engineered structures. These systems are particularly important in applications where localized or sequential component failures can threaten overall system performance, such as in telecommunication networks, oil and gas pipelines, particle accelerators, and large-scale computing architectures. These systems are typically classified according to the physical arrangement of components (linear or circular) and the criteria for system operability. In a linear consecutive k-out-of-n:G system, components are arranged in a fixed sequence, and the system is considered functional only if at least *k* consecutive components are operating correctly. This distinguishes them from classical k-out-of-n:G systems, where the functioning of any *k* components, regardless of their position, is sufficient for operability. Both series and parallel systems arise as limiting cases of this broader framework: when k = n, the structure reduces to a strict series system, whereas when *k* = 1, it behaves as a parallel system in which a single functioning component suffices to sustain operation. The linear consecutive version captures spatial dependence and localized vulnerability, which makes it particularly relevant for modern applications where failures often occur in adjacent segments. To illustrate this distinction, Figure 1 presents a standard 2-out-of-3:G system and its linear consecutive counterpart.

The structural and probabilistic properties of consecutive systems have been the subject of extensive study, yet their treatment from an information-theoretic perspective remains comparatively limited. Classical measures of uncertainty, such as Shannon entropy, together with their generalizations, including Rényi and Tsallis entropy, have long served as effective tools for quantifying variability and complexity in probability distributions. Beyond their theoretical significance, Shannon and Tsallis entropies have found diverse applications across scientific and engineering fields. Shannon entropy, as the cornerstone of information theory, has long been applied in data compression, coding, communication networks, and system reliability assessment, where it quantifies uncertainty and information flow. More recently, it has been employed in reaction–diffusion processes and mixing efficiency studies in engineering and environmental sciences. Tsallis entropy, introduced as a non-additive generalization of Shannon entropy, has been extensively utilized in statistical mechanics, complex systems, and physics, as well as in practical areas such as medical imaging, remote sensing, and pattern recognition through entropy-based segmentation and thresholding methods. It has also been applied to finance and random network modeling, where non-extensive statistics provide more realistic descriptions of uncertainty. These developments highlight the broad practical relevance of entropy-based measures, providing motivation for our present contribution, which extends the use of CRTE to the study of linear consecutive k-out-of-n:G systems. More recently, the cumulative residual entropy and its variants have emerged as dynamic measures tailored to reliability and lifetime analysis, offering insights beyond those provided by variance-based approaches.

However, the role of CRTE in the study of consecutive k-out-of-n:G systems has not yet been systematically examined, despite its considerable potential for advancing our understanding of stochastic ordering, uncertainty quantification, and entropy-based system characterization. We address this gap by developing a comprehensive CRTE-based framework for the analysis of linear consecutive k-out-of-n:G systems. By uniting reliability modeling with entropy-driven approaches, the study provides a fresh perspective that both deepens theoretical understanding and demonstrates the versatility of CRTE as a tool for bounding analysis, distributional characterization, and statistical inference in complex system structures.

The theoretical underpinnings and practical relevance of k-out-of-n system models have been extensively investigated in the literature [1,2,3,4,5,6,7], underscoring their central role in the reliability analysis of complex systems. Among these, linear consecutive k-out-of-n:G systems have attracted particular attention, especially under the condition 2k≥n, due to their analytical tractability and structural simplicity. Within this framework, the lifetime of the i-th component is modeled by a random variable $X_{i}$, i=1,2,…n. These lifetimes are typically assumed to be independent and identically distributed (i.i.d.), governed by a common probability density function (pdf) h(x) and cumulative distribution function (cdf) H(x). The overall system lifetime, denoted by $T_{k|n:G}$, represents the time until the first occurrence of *k* consecutive functioning components. As shown by Eryilmaz [8], when the condition 2 k ≥ n holds, the survival function of the system admits a closed-form expression:

(1) $${\bar{H}}_{k|n:G}(x)=(n-k+1){\bar{H}}^{k}(x)-(n-k){\bar{H}}^{k+1}(x),$$

x>0, where $\bar{H}(x)=P(X>x)$ denotes the survival function associated with X.

Although the probabilistic properties of consecutive systems are well understood, their characterization from an information-theoretic standpoint remains relatively undeveloped. Classical entropy measures such as Shannon, Rényi, and Tsallis entropy have proven effective in describing variability and complexity in probability distributions. Building on these, the CRE and its generalizations have emerged as dynamic tools for lifetime and reliability analysis, capturing aspects of uncertainty not reflected in variance-based metrics. Although the CRTE shows considerable potential for advancing our understanding of stochastic ordering, uncertainty quantification, and entropy-based system characterization, it has not yet been systematically investigated in the context of linear consecutive k-out-of-n:G systems. To the best of our knowledge, no prior study has addressed the role of CRTE in such systems, despite their practical importance in reliability engineering. To bridge this gap, the present work develops a comprehensive CRTE framework for analyzing linear consecutive k-out-of-n:G systems. Our contributions include the derivation of analytical bounds, the establishment of characterization theorems and ordering results, and the introduction of a novel nonparametric test for dispersive ordering. The proposed test is rigorously supported by asymptotic theory and validated through extensive simulation experiments.

In recent years, the quantification of uncertainty in probability distributions has attracted considerable interest due to its theoretical appeal and practical implications. A cornerstone of this research is Shannon’s differential entropy, which is defined for a non-negative continuous random variable X with probability density function h(x) as H(X)=−E[log h(X)], where log(⋅) means natural logarithm. This classical entropy measure has inspired a variety of generalizations aimed at capturing different aspects of information complexity and uncertainty. These include the generalized entropy (GE) of order α, introduced as:

$$H_{\alpha }\left(X\right)=\frac{1}{\alpha -1}\int_{0}^{\infty }\left[h\left(x\right)-h^{\alpha }\left(x\right)\right]dx,$$

$\alpha \in \Omega =\left(0,1\right)\cup \left(1,\infty \right),$ which reduces to Shannon entropy in the limiting case as α→1 [9]. This family of entropies includes Shannon entropy as a special case, given by:

$$H\left(X\right)=\underset{\alpha \to 1}{lim}\;H_{\alpha }\left(X\right).$$

The GE of order α is particularly well-suited for nonextensive systems, where the distribution of a random variable is expressed through the escort distribution of order *q*, defined as the *q*th power of another distribution. Such discrete escort distributions frequently arise in areas including nonextensive statistical mechanics, source coding (see Bercher [10]), and nanothermodynamics (see Vakili-Nezhaad and Mansoori [11]). Building on this foundation, Rajesh and Sunoj [12] introduced the CRTE by substituting the pdf h(x) with the survival function $\bar{H}(x)$ in the generalized entropy formula. This yields

(2) $$\xi_{\alpha }(X)=\frac{1}{\alpha -1}\int_{0}^{\infty }\left[\bar{H}(x)-{\bar{H}}^{\alpha }(x)\right]dx$$

(3) $$=\int_{0}^{1}\frac{\chi_{\alpha }(u)}{h\left(H^{-1}(u)\right)}du,$$

where $F^{-1}(u)=inf\{x;H(x)\geq u\}$ is the quantile function of H(x) and

(4) $$\chi_{\alpha }\left(u\right)=\frac{1}{\alpha -1}\left((1-u)-(1-u{)}^{\alpha }\right),$$

0<u<1, for α∈Ω. This family includes the cumulative residual entropy (CRE) as a special case as

$$\xi (X)=\underset{\alpha \to 1}{lim}\;\xi_{\alpha }(X)=-\int_{0}^{\infty }\;\bar{H}(x)log\;\bar{H}(x)dx.$$

The foundational CRE was extensively examined by Rao et al. [13]. Subsequent advancements introduced dynamic extensions, as studied by Asadi and Zohrevand [14], and further elaborated by Navarro et al. [15]. The relevance of this entropy measure to reliability engineering was later emphasized by Toomaj et al. [16]. Differential entropy quantifies the divergence of the probability density function h(x) from a uniform distribution. It reflects the level of uncertainty associated with using h(x) compared to the inherent uncertainty of a uniform distribution. Furthermore, for an atom distribution F where X = c almost surely, we find that $\xi_{\alpha }\left(X\right)=0$. This scenario represents the most informative case, characterized by minimal uncertainty. Consequently, $\xi_{\alpha }\left(X\right)=0$ can serve as a measure to assess how closely X approaches to an atom distribution, functioning as a dispersion metric. In the realm of continuous distributions, Shannon differential entropy quantifies the divergence of the probability density function *h*(*x*) from a uniform distribution. One of the benefits of CRTE is its applicability to both continuous and discrete distributions. In contrast, the generalized entropy of order α and other entropy measures based on probability density functions are only relevant when the pdf exists. Additionally, the CRTE assesses the dispersion of a random variable, much like variance, making it particularly useful in relation to variance as pointed out by Toomaj and Agh Atabay [17]. Another advantage of the CRTE is its relationship with the proportional hazards (PH) model. It links the Bayes risks associated with the mean excess of a random variable to the PH distribution, as well as to the Bayes risks of the Fisher information of the equilibrium distribution within the PH model, as explored by Asadi et al. [18].

Two main research areas on system comparison have emerged in reliability theory over the last two decades. One, initiated by Samaniego [19] and extended by Kochar et al. [20], uses mixture representations of lifetime distributions for coherent systems with homogeneous components. The other applies Shannon differential entropy to compare systems based on uncertainty and information in lifetime prediction, as seen in [21]. Toomaj and Doostparast [22] recently merged these ideas, and since then, several authors have explored the information properties of engineering systems. For example, Toomaj et al. investigated the CRE properties of coherent and mixed systems. Asadi et al. [23] proposed Jensen–Shannon information criteria to compare coherent systems, ranking them based on design signatures and providing useful results. Kayid and Alshehri [24] studied the CRE of the remaining lifetime of a mixed system with n components, assuming all are operational at time t, deriving expressions, limits, and order properties using the system signature; see also Alomani and Kayid [25], Shrahili and Kayid [26], Kayid and Shrahili [27] and references therein. Recently, consecutive systems have attracted interest regarding their information properties. Kayid and Alshehri [28] provided results on Shannon differential entropy for consecutive k-out-of-n:G systems, including expressions, bounds, and nonparametric estimation for practical application, see also Kayid and Shrahili [29]. However, the role of CRTE in consecutive systems remains largely unstudied. This paper seeks to bridge this gap by providing a comprehensive information-theoretic analysis of CRTE in the context of consecutive k-out-of-n:G systems, a setting not previously examined in the literature, with the objective of uncovering deeper insights into its structural properties, stochastic behavior, and practical applicability to reliability modeling.

The main contributions of this work can be summarized as follows: (i). We derive a general analytical expression for the CRTE of consecutive k-out-of-n:G systems based on an arbitrary continuous distribution; (ii). we establish new stochastic ordering results and informative bounds that characterize the uncertainty of these systems; (iii). we present a set of characterization theorems that link CRTE to fundamental reliability and information measures; and (iv). we propose a novel nonparametric test for dispersive ordering, supported by asymptotic theory and validated through Monte Carlo simulations.

The remainder of this paper is organized as follows. Section 2 develops the CRTE expression and investigates stochastic ordering and bounds. Section 3 establishes characterization results. Section 4 introduces the nonparametric dispersive-ordering test and provides supporting simulations. Section 5 concludes the study by summarizing the contributions and outlining directions for future research.

## 2. Linear Consecutive k-out-of-n:G Systems: Structure and CRTE Framework

This section is divided into two parts. In the first part, an explicit mathematical formulation for the CRTE of a consecutive k-out-of-n:G system is derived, and its behavior under various stochastic ordering relations is examined. The second part is devoted to establishing analytical bounds for the CRTE, which serve as valuable tools for evaluating and comparing the uncertainty characteristics of such systems.

### 2.1. Analytical Expression of CRTE and Stochastic Ordering Results

A precise expression for the CRTE of a consecutive k-out-of-n:G system with lifetime $T_{k|n:G}$ is derived, assuming that the component lifetimes follow a common continuous distribution function H. By applying the probability integral transformation $U_{k|n:G}=H\left(T_{k|n:G}\right)$, a useful formula is obtained. Under this transformation, the component lifetimes $U_{i}=H\left(X_{i}\right)$, for i=1,…,n, become independent and identically distributed (i.i.d.) uniform random variables on the interval [0,1]. As shown in (1), the survival function of $U_{k|n:G}$, under the condition 2k≥n, is given by

(5) $${\bar{G}}_{k|n:G}(u)=(n-k+1)(1-u{)}^{k}-(n-k)(1-u{)}^{k+1},$$

for 0<u<1. It follows that for 0<u<1

(6) $$g_{k|n:G}(u)=k(n-k+1)(1-u{)}^{k-1}-(k+1)(n-k)(1-u{)}^{k}.$$

Based on our previous findings, we now state the following theorem. Since its proof closely follows Theorem 1 of Kayid and Shrahili [29], it is omitted for brevity. Let us define $C_{X}$ as the class of consecutive systems that share a common i.i.d. component lifetime distribution, characterized by the pdf h(x) and cdf H(x).

**Theorem** **1.** *If* $T_{k|n:G}\in C_{X}$*, then the CRTE of* $T_{k|n:G}$ *for* 2k≥n*, can be expressed as*

(7) $$\xi_{\alpha }\left(T_{k|n:G}\right)=\int_{0}^{1}\frac{\chi_{\alpha }\left({\bar{G}}_{k|n:G}(u)\right)}{h\left(H^{-1}(u)\right)}du,\;for\;all\;\alpha \in \Omega ,\;$$

*where* $\chi_{\alpha }(x)$ *and* ${\bar{G}}_{k|n:G}(u)$ *are defined in (4) and (5), respectively.*

*We now present a sufficient condition for the existence of the CRTE of a consecutive* k*-out-of-*n*:G system, denoted as* $\xi_{\alpha }\left(T_{k|n:G}\right)$*, in the subsequent theorem.*

**Theorem** **2.** *Let* $T_{k|n:G}\in C_{X}$*. Then* $\xi_{\alpha }\left(T_{k|n:G}\right)<\infty$ *for all* α>0 *and fixed* n*, provided that* $E\left(X^{p}\right)<\infty$*, for some*

$$p\left\{\begin{array}{ll} \geq 1 & \alpha >1\\ >\frac{1}{\alpha } & 0<\alpha <1 \end{array}.\right.$$

**Proof.**  For α>1, it is straightforward to observe that $\frac{x-x^{\alpha }}{\alpha -1}\leq \frac{x}{\alpha -1}$, for 0≤x≤1. Combining this with the fact that $-(n-k){\bar{H}}^{k+1}(x)\leq 0$, and ${\bar{H}}^{k}\left(x\right)\leq \bar{H}\left(x\right),\;\forall \;k\geq 1$ and x>0, and recalling (1) and (2), we obtain

$$\begin{array}{ll} \xi_{\alpha }\left(T_{k|n:G}\right) & \leq \frac{1}{\alpha -1}\int_{0}^{\infty }\;{\bar{H}}_{k|n:G}(x)dx\leq \frac{\left(n-k+1\right)}{\alpha -1}\int_{0}^{\infty }\;{\bar{H}}^{k}(x)dx\\ & \leq \frac{\left(n-k+1\right)E\left(X\right)}{\alpha -1}\leq \frac{\left(n-k+1\right){\left(E\left(X^{p}\right)\right)}^{\frac{1}{p}}}{\alpha -1}<\infty , \end{array}$$

where the fourth inequality is derived using Lyapunov’s inequality. Now, consider the case when 0<α<1, it holds that $\frac{x^{\alpha }-x}{1-\alpha }\leq \frac{x^{\alpha }}{1-\alpha }$, for 0≤x≤1. Additionally, since ${\bar{H}}^{\alpha k}(x)\leq {\bar{H}}^{\alpha }(x)$, for k≥1 and 0<α<1, it follows that

$$\begin{array}{ll} \xi_{\alpha }\left(T_{k|n:G}\right) & \leq \frac{1}{1-\alpha }\int_{0}^{\infty }\;{\bar{H}}_{k|n:G}^{\alpha }(x)dx\leq \frac{(n-k+1{)}^{\alpha }}{1-\alpha }\int_{0}^{\infty }\;{\bar{H}}^{\alpha }(x)dx\\ & \leq \frac{(n-k+1{)}^{\alpha }{\left(E\left(X^{p}\right)\right)}^{\alpha }}{1-\alpha }\int_{0}^{\infty }\;\frac{1}{x^{\alpha p}}\;dx<\infty , \end{array}$$

where the last inequality is finite provided that p>1/α, thereby completing the proof. □

In the following theorem, we provide an alternative representation for $\xi_{\alpha }\left(T_{k|n:G}\right)$ using Newton’s generalized binomial theorem.

**Theorem** **3.** *If* $T_{k|n:G}\in C_{X}$*, for* 2k≥n*, we have*

$$\begin{array}{ll} \xi_{\alpha }\left(T_{k|n:G}\right) & =\frac{1}{\alpha -1}\left(\left(n-k+1\right)E\left(X_{1:k}\right)-\left(n-k\right)E\left(X_{1:k+1}\right)\right)\;\; \end{array}$$

(8) −(n−k+1)αα−1∑i=0∞  αin−kk−n−1ii+αk+1E1hH−11−Zi,k,α,

*where* $Z_{i,k,\alpha }∼Beta(i+\alpha k+1,1)$*.*

**Proof.**  Recalling (1), the first term on the right-hand side of (8) is straightforward. For the second term, let A=(n−k+1) and B=(n−k). By referring to (5) and applying the substitution u=H(x), we obtain

∫0∞ H¯k∣n:Gα(x)dx=∫01 G¯k∣n:GαuhH−1udu=∫01 (1−u)αk(A−B(1−u))αhH−1(u)du=Aα∫01 zαk1−BAzαhH−1(1−z)dz,(taking z=1−u)=Aα∑i=0∞  αiBAi(−1)i∫01 zi+αkhH−1(1−z)dz=(n−k+1)α∑i=0∞  αin−kk−n−1ii+αk+1E1hH−11−Zi,k,α,

where the third equality follows directly from Newton’s generalized binomial series $(1-x{)}^{\alpha }=$ ∑i=0∞ αi(−1)ixi, with αi=α(α−1)⋯(α−i+1)i!, which converges for  |x|<1. Since 0<z<1 and B<A, we have $0<\frac{B}{A}z<\frac{B}{A}<1,$ ensuring the convergence of the series. Therefore, the result holds. □

Using the representation in Equation (7), we present an illustrative example.

**Example** **1.** *Consider a linear consecutive 2-out-of-4:G system with a lifetime*

$$T_{2|4:G}=max\left(min\left(X_{1},X_{2}\right),min\left(X_{2},X_{3}\right),min\left(,X_{3},X_{4}\right)\right).$$

*Assume that the component lifetimes* $X_{i}$*(*i=1,2,…,4*) are i.i.d. random variables following a common Rayleigh distribution with the survival function as*

$$\bar{H}\left(x\right)=e^{-\frac{x^{2}}{2\sigma^{2}}},\;x>0,\sigma >0.\;$$

**Figure 2:**
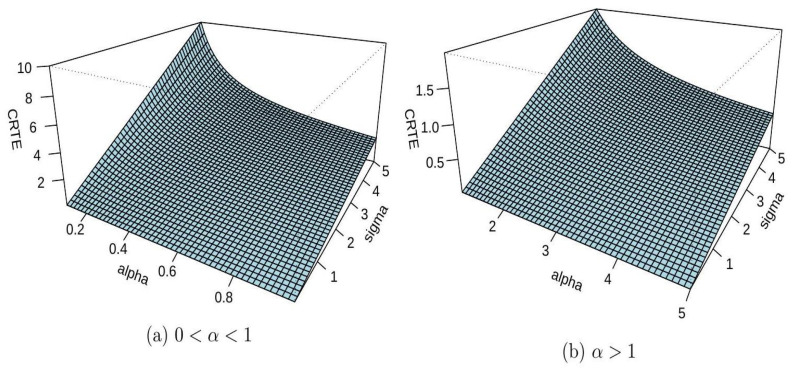
The plot of $\xi_{\alpha }\left(T_{2|4:G}\right)$ with respect to α as demonstrated in Example 1.

*Note that the Rayleigh distribution corresponds to a chi distribution with two degrees of freedom. It is not hard to see that* $h\left(H^{-1}(u)\right)=\frac{1-u}{\sigma }\sqrt{-2log(1-u)}$ *for all* 0<u<1*. Recalling (8), it allows us to derive the following expression*

$$\xi_{\alpha }\left(T_{2|4:G}\right)=\sigma \int_{0}^{1}\;\frac{\chi_{\alpha }\left({\bar{G}}_{k|n:G}(u)\right)}{(1-u)\sqrt{-2log(1-u)}}du,\;$$

for all α∈Ω. *Because deriving an explicit analytical expression is difficult, a computational approach is used to examine the relationship between* $\xi_{\alpha }\left(T_{2|4:G}\right),\alpha$*, and the Rayleigh distribution parameter* σ*. The analysis highlights how* σ *influences the CRTE of the consecutive 2-out-of-4:G system. Figure 2 presents the results, illustrating the interaction between* $\xi_{\alpha }\left(T_{2|4:G}\right),\;\sigma$ *and* α*. The system’s uncertainty, as measured by the CRTE, initially decreases and then increases as the scale parameter* σ *increases. These findings emphasize the significant impact of the Rayleigh distribution parameter* σ *on both the CRTE and the uncertainty of the consecutive 2-out-of-4:G system.*

The following theorem investigates the conditions for preserving dispersive ordering under the CRTE of the consecutive k-out-of-n:G systems. Let X and Y be two random variables with cdfs $H_{X}(x)$ and $H_{Y}(x)$, respectively. We recall that X is smaller than Y in the dispersion order (denoted by $X\leq_{\mathrm{disp}}Y$) if $H_{X}^{-1}(v)-H_{X}^{-1}(u)\leq H_{Y}^{-1}(v)-H_{Y}^{-1}(u),0<u\leq v<1$. On the other hand, $X\leq_{disp}Y$ if and only if

$$H_{Y}^{-1}(u)-H_{X}^{-1}(u)\;\mathrm{is}\;\mathrm{increasing}\;\mathrm{in}\;u\in (0,1),$$

where $H_{X}^{-1}$ and $H_{Y}^{-1}$ are left continuous inverses of $H_{X}$ and $H_{Y}$, respectively (see Shaked and Shanthikumar [30]).

**Theorem** **4.** *Let* $T_{k|n:G}^{X}\in C_{X}$ *and* $T_{k|n:G}^{Y}\in C_{Y}$*. If* $X\leq_{\mathrm{disp}}Y$*, then* $\xi_{\alpha }\left(T_{k|n:G}^{X}\right)\leq \xi_{\alpha }\left(T_{k|n:G}^{Y}\right)$ *for all* α∈Ω.

**Proof.**  By changing the variable to u=H(x), using (1) and (2), we obtain

$$\begin{array}{ll} \xi_{\alpha }\left(T_{k|n:G}^{X}\right) & =\int_{0}^{\infty }\;\chi_{\alpha }\left({\bar{H}}_{k|n:G}(x)\right)dx=\int_{0}^{\infty }\;\chi_{\alpha }\left({\bar{G}}_{k|n:G}\left(u\right)\right)dH_{X}^{-1}\left(u\right),\;for\;all\;\alpha \in \Omega . \end{array}$$

A similar argument applies to $\xi_{\alpha }\left(T_{k|n:G}^{Y}\right)$. Given the assumption that $X\leq_{\mathrm{disp}}Y$, we have

$$d\left[H_{Y}^{-1}(u)-H_{X}^{-1}(u)\right]\geq 0.$$

This yield

(9) $$\xi_{\alpha }\left(T_{k|n:G}^{Y}\right)-\xi_{\alpha }\left(T_{k|n:G}^{X}\right)=\int_{0}^{\infty }\;\chi_{\alpha }\left({\bar{G}}_{k|n:G}(u)\right)d\left[H_{Y}^{-1}(u)-H_{X}^{-1}(u)\right]\geq 0,$$

for all α∈Ω, by noting that $\chi_{\alpha }\left({\bar{G}}_{k|n:G}(u)\right)\geq 0$, for all 0<u<1. Hence, the theorem.

An advantage of the above theorem is that the random lifetimes do not need to be absolutely continuous. If $\lambda (x)=\frac{h(x)}{\bar{H}(x)}$, for x>0, denotes the hazard rate function of X, then X is said to have a decreasing failure rate (DFR) property if λ(x) is a decreasing function of x. It is well known that if

(10) $$X\leq_{hr}Y\;\mathrm{and}\;\mathrm{either}\;X\;\mathrm{or}\;Y\;\mathrm{is}\;\mathrm{DFR}\;⟹X\leq_{disp}Y.$$

From implication (10) and Theorem 4, we can derive the following corollary. □

**Corollary** **1.** *Under the conditions of Theorem 4, if* $X\leq_{hr}Y$ *and either* X *or* Y *is DFR, then* $\xi_{\alpha }\left(T_{k|n:G}^{X}\right)\leq \xi_{\alpha }\left(T_{k|n:G}^{Y}\right)$ *for all* α∈Ω.

As an application of Corollary 1, consider the following example.

**Example** **2.** *Consider two consecutive 4-out-of-5:G systems with lifetimes* $T_{4|5:G}^{X}$ *and* $T_{4|5:G}^{Y}$*. The first system,* $T_{4|5:G}^{X}$*, has i.i.d. component lifetimes* $X_{1},X_{2},X_{3},X_{4},X_{5}$ *that follow the Makeham distribution with the survival function* ${\bar{H}}_{X}(x)=e^{-2x+e^{-x}-1}$ *for* x>0 *. The second system,* $T_{4|5:G}^{Y}$*, consists of i.i.d. component lifetimes* $Y_{1},Y_{2},Y_{3},Y_{4}$ *that follow an exponential distribution with* $cdf\;{\bar{H}}_{Y}(x)=e^{-x}$ *for* x>0*. The hazard rate functions are* $\lambda_{X}(x)=2-e^{-x}$ *and* $\lambda_{Y}(x)=1$*, indicating that* $\lambda_{X}(x)>\lambda_{Y}(x)$ *for* x>0*, i.e.,* $X\leq_{hr}Y$*. Since the exponential distribution has the DFR property, consequently, by Corollary 1, we have* $\xi_{\alpha }\left(T_{4|5:G}^{X}\right)\leq \xi_{\alpha }\left(T_{4|5:G}^{Y}\right)$*, implying that the uncertainty associated with* $T_{4|5:G}^{X}$ *is less than or equal to that of* $T_{4|5:G}^{Y}$ *in terms of the CRTE measure.*

The next theorem offers an interesting result on the characterization of dispersive order.

**Theorem** **5.** *Let* $T_{k|n:G}^{X}\in C_{X}$ *and* $T_{k|n:G}^{Y}\in C_{Y}$*. If* $X\leq_{\mathrm{disp}}Y$ *and if*

(11) $$\xi_{\alpha }\left(T_{k|n:G}^{X}\right)=\xi_{\alpha }\left(T_{k|n:G}^{Y}\right),\;for\;\;\alpha \in \Omega ,\;$$

*then*X *and* Y *have the same distribution but for a location change.*

**Proof.**  Assuming (11) holds for some α∈Ω, we obtain from (9) that

$$\xi_{\alpha }\left(T_{k|n:G}^{Y}\right)-\xi_{\alpha }\left(T_{k|n:G}^{X}\right)==\int_{0}^{\infty }\;\chi_{\alpha }\left({\bar{G}}_{k|n:G}(u)\right)d\left[H_{Y}^{-1}(u)-H_{X}^{-1}(u)\right]=0.\;$$

Since $X\leq_{\mathrm{disp}}Y$, it follows that $H_{Y}^{-1}(u)-H_{X}^{-1}(u)$ is increasing in u. We claim the difference is a constant c, for all u∈[0,1]. Assume, for contradiction, that there exists an interval (a,b)⊂[0,1] where $H_{Y}^{-1}(u)-H_{X}^{-1}(u)$ is not constant. Then,

$$0=\int_{0}^{1}\;\chi_{\alpha }\left({\bar{G}}_{k|n:G}(u)\right)d\left[H_{Y}^{-1}(u)-H_{X}^{-1}(u)\right]\geq \int_{a}^{b}\;\chi_{\alpha }\left({\bar{G}}_{k|n:G}(u)\right)d\left[H_{Y}^{-1}(u)-H_{X}^{-1}(u)\right]>0.$$

This indicates a contradiction. Thus, $H_{Y}^{-1}(u)-H_{X}^{-1}(u)=c$ is a constant for all u∈[0,1], implying that X and Y have identical distributions but for a location shift. Thus, identical CRTEs imply the distributions differ only by a shift.

The following theorem establishes the conditions under which the location-independent riskier order is preserved in consecutive systems. We recall that X is less than or equal to Y in the location-independent riskier order (denoted $X\leq_{lir}Y$) if

$$\int_{0}^{H_{X}^{-1}\left(p\right)}\;H_{X}\left(x\right)dx\leq \int_{0}^{H_{Y}^{-1}\left(p\right)}\;H_{Y}\left(x\right)dx,p\in \left(0,1\right).$$

Let us revisit the integrated distribution function of a random variable Z whose cdfH is denoted by

$$\eta_{Z}(x)=\int_{0}^{x}\;H(z)dz,x>0.$$

It is shown that (see Landsberger and Meilijson [31])

$$X\leq_{lir}Y⟺\eta_{Y}^{-1}(x)-\eta_{X}^{-1}(x)\;\mathrm{is}\;\mathrm{increasing}\;\mathrm{in}\;x>0.$$

We now present the following theorem. Its proof closely resembles Theorem 4 of Kayid and Shrahili [29] and is therefore omitted. □

**Theorem** **6.** *Let* $T_{k|n:G}^{X}\in C_{X}$ *and* $T_{k|n:G}^{Y}\in C_{Y}$*. If* $X\leq_{\mathrm{lir}}Y$*, and*

$$\frac{\chi_{\alpha }\left({\bar{G}}_{k|n:G}(t)\right)}{t},0\leq t\leq 1,$$

*is a decreasing function of* t*, then* $\xi_{\alpha }\left(T_{k|n:G}^{X}\right)\leq \xi_{\alpha }\left(T_{k|n:G}^{Y}\right)$ *for all* α∈Ω.

### 2.2. Practical Bounds for CRTE in Reliability and Uncertainty Quantification

In many practical situations, obtaining a closed-form expression for the CRTE of consecutive systems can be challenging, particularly when the underlying lifetime distributions are analytically complex or when the system comprises a large number of components. In such cases, establishing informative and tractable bounds is essential to effectively characterize the CRTE. Motivated by this challenge, we derive a set of bounds that offer theoretical insight into the behavior of CRTE in consecutive k-out-of-n:G systems. The results are presented in the following theorem. The proof parallels the techniques employed in Theorems 5 and 6 of Kayid and Shrahili [29]; it is omitted for conciseness.

**Theorem** **7.** *(i). For* 2k≥n*, the CRTE of* $T_{k|n:G}$ *is bounded as follows:*

$$B_{1,\alpha }\xi_{\alpha }\left(X_{1}\right)\leq \xi_{\alpha }\left(T_{k|n:G}\right)\leq B_{2,\alpha }\xi_{\alpha }\left(X_{1}\right),$$

*where* $B_{1,\alpha }=\underset{u\in (0,1)}{inf}\;\frac{\chi_{\alpha }\left({\bar{G}}_{k|n:G}(u)\right)}{\chi_{\alpha }(u)},B_{2,\alpha }=\underset{u\in (0,1)}{sup}\;\frac{\chi_{\alpha }\left({\bar{G}}_{k|n:G}(u)\right)}{\chi_{\alpha }(u)}$ *for* α∈Ω.

*(ii). Let* $T_{k|n:G}\in C_{X}$*. If* $m=\underset{x>0}{inf}\;h(x)$ *and* $M=\underset{x>0}{sup}\;h(x)$*, then*

$$\frac{\xi_{\alpha }\left(U_{k|n:G}\right)}{M}\leq \xi_{\alpha }\left(T_{r|n:G}\right)\leq \frac{\xi_{\alpha }\left(U_{k|n:G}\right)}{m},$$

*where* $\xi_{\alpha }\left(U_{k|n:G}\right)=\int_{0}^{1}\;\chi_{\alpha }\left({\bar{G}}_{k|n:G}(u)\right)du$ *for* α∈Ω.

Part (i) of Theorem 7 establishes that the CRTE of the system is bounded above by the common CRTE of its individual components. Part (ii) provides additional, computationally convenient bounds that depend on the minimum and maximum values of the probability density function, as well as on the function $\xi_{\alpha }\left(U_{k|n:G}\right)$, which denotes the CRTE of a consecutive k-out-of-n:G system with uniformly distributed components over the interval (0,1). Notably, setting the lower bound parameter m=0 implies that no finite upper bound exists, while an upper bound of M=∞ eliminates the lower bound. The following example illustrates the practical application of Theorem 7 to a consecutive k-out-of-n:G system.

**Example** **3.** *Consider a linear consecutive 5-out-of-10:G system with a lifetime defined as* $T_{5|10:G}=max\left(X_{\left[1:5\right]},X_{\left[2:6\right]},\ldots ,X_{\left[5:10\right]}\right)$*, where* $X_{\left[j:m\right]}=min\left(X_{j},\ldots ,X_{m}\right),$ *for* 1≤j<m≤10*. Assume the component lifetimes follow a Gompertz distribution with the following survival function*

$${\bar{H}}_{X}(x)=e^{-2\left(e^{x}-1\right)},x>0.$$

*It is not hard to see that* m=0 *and* M=2*. Moreover, the CRTE of the Gompertz distribution is given by*

$$\begin{array}{ll} \xi_{\alpha }(X) & =\frac{1}{\alpha -1}\int_{0}^{\infty }\;\left[e^{-2\left(e^{x}-1\right)}-e^{-2\alpha \left(e^{x}-1\right)}\right]dx\\ & =\frac{1}{\alpha -1}\left[e^{2}E_{1}(2)-e^{2\alpha }E_{1}(2\alpha )\right],\;for\;all\;\alpha \in \Omega ,\; \end{array}$$

*where*

$$E_{1}\left(x\right)=\int_{x}^{\infty }\;\frac{e^{-t}}{t}dt,$$

 *stands for the exponential integral. By merging the bounds from Theorem 7, we find that the CRTE of the consecutive system is bounded as* $L_{\alpha }\left(T_{5|10:G}\right)\leq \xi_{\alpha }\left(T_{5|10:G}\right)\leq U_{\alpha }\left(T_{5|10:G}\right)$*, where*

$$L_{\alpha }\left(T_{5|10:G}\right)=0.5\xi_{\alpha }\left(U_{k|n:G}\right),\;\mathrm{and}\;U_{\alpha }\left(T_{5|10:G}\right)=\frac{B_{2,\alpha }}{\alpha -1}\left[e^{2}E_{1}(2)-e^{2\alpha }E_{1}(2\alpha )\right].$$

Table 1 displays the values of these expressions. The bounds in Part (ii) of Theorem 7 are significant, straightforward, and beneficial for practical applications.

In the next theorem, we establish bounds for the CRTE of consecutive k-out-of-n:G systems. These bounds are directly connected to the hazard rate function of the component lifetimes. To this aim, we observe that

(12) $$\begin{array}{ll} \xi_{\alpha }\left(X\right) & =\frac{1}{\alpha -1}\int_{0}^{\infty }\;\left[\bar{H}\left(x\right)-{\bar{H}}^{\alpha }\left(x\right)\right]dx\\ & =\frac{1}{\alpha -1}\int_{0}^{\infty }\;\frac{\bar{H}\left(x\right)}{h\left(x\right)}\left(1-{\bar{H}}^{\alpha -1}\left(x\right)\right)h\left(x\right)dx=\frac{1}{\alpha }E\left(\frac{1}{\lambda \left(X_{\alpha }\right)}\right),\; \end{array}$$

where $X_{\alpha }$ has the pdf

$$h_{\alpha }(x)=\frac{\alpha }{\alpha -1}h(x)\left(1-{\bar{H}}^{\alpha -1}(x)\right),x>0,$$

for all α∈Ω and λ(x) denotes the hazard rate function of X. This representation of the CRTE in terms of the hazard rate function of $X_{\alpha }$ provides a crucial foundation for deriving the subsequent bounds for the CRTE of consecutive k-out-of-n:G systems.

**Theorem** **8.** *Let* $T_{k|n:G}\in C_{X}$ *having the common failure rate function* λ(x)*. For all* 2k≥n*, we have*

$$\frac{1}{k\alpha }E\left(\frac{1}{\lambda \left(T_{k|n:G,\alpha }\right)}\right)\leq \xi_{\alpha }\left(T_{k|n:G}\right)\leq \frac{1}{(2k-n)\alpha }E\left(\frac{1}{\lambda \left(T_{k|n:G,\alpha }\right)}\right),$$

*where* $T_{k|n:G,\alpha }$ *has the pdf* $h_{k|n:G,\alpha }(x)=\frac{\alpha }{\alpha -1}h_{k|n:G}(x)\left(1-{\bar{H}}_{k|n:G}^{\alpha -1}(x)\right)$*, for* x>0 *and* α∈Ω.

**Proof.**  It is easy to see that the hazard rate function of $T_{k|n:G}$ can be expressed as $\lambda_{k|n:G}(x)=\psi_{k,n}(\bar{H}(x))\lambda (x)$, where

$$\psi_{k,n}(z)=\frac{k\left(n-k+1\right)-\left(k+1\right)\left(n-k\right)z}{\left(n-k+1\right)-\left(n-k\right)z},0<z<1.$$

Since $\psi_{k,n}^{\prime }(z)<0$ for 2k≥n and 0<z<1, it follows that $\psi_{k,n}(z)$ is a monotonically decreasing function of z. Given that $\psi_{k,n}(0)=k$ and $\psi_{k,n}(1)=2k-n$, we have $2k-n\leq \psi_{k,n}(\bar{H}(x))\leq k$ for $0<\bar{H}(x)<1$, which implies that $\left(2k-n)\lambda (x)\leq \lambda_{k|n:G}(\bar{H}(x))\leq k\lambda (x\right)$, for x>0. Combining this result with Equation (12) completes the proof. □

The subsequent theorem is valid under the condition that the expected value of the reciprocal of the squared hazard rate function of X is finite.

**Theorem** **9.** *Under the conditions of Theorem 8 such that* $E\left(\frac{1}{\lambda^{2}(X)}\right)<\infty$*, for* 2k≥n *and* α>1*, the following inequalities hold:*

$$\xi_{\alpha }\left(T_{k|n:G}\right)\leq \frac{\sqrt{\Omega_{r,n,\alpha }E\left(\frac{1}{\lambda^{2}(X)}\right)}}{\alpha -1},$$

*where* $\Omega_{k,n,\alpha }=\int_{0}^{1}\;{\left[\frac{{\bar{G}}_{k|n:G}(u)-{\bar{G}}_{k|n:G}^{\alpha }(u)}{1-u}\right]}^{2}du$*. Conversely, when* 0<α<1*, the inequality is reversed.*

**Proof.**  Let α>1. The pdf of $T_{k|n:G}$ can be rewritten as $h_{k|n:G}(x)=h(x)g_{k|n:G}(H(x))$, where $g_{k|n:G}(u)$, defined in (6) while its failure rate function is given by

$$\lambda_{k|n:G}\left(x\right)=\lambda \left(x\right)\frac{\bar{H}\left(x\right)g_{k|n:G}\left(H\left(x\right)\right)}{{\bar{H}}_{k|n:G}\left(x\right)},for\;x>0.$$

Consequently, by (12) and using Cauchy–Schwarz inequality, we obtain

$$\begin{array}{ll} \int_{0}^{\infty }\;\frac{h_{k|n:G}(x)\left(1-{\bar{H}}_{k|n:G}^{\alpha -1}(x)\right)}{\lambda_{k|n:G}(x)}dx & =\int_{0}^{\infty }\;\frac{\sqrt{h(x)}\sqrt{h(x)}{\bar{H}}_{k|n:G}(x)\left(1-{\bar{H}}_{k|n:G}^{\alpha -1}(x)\right)}{\lambda (x)\bar{H}(x)}dx\\ & \leq {\left(\int_{0}^{\infty }\;\frac{h(x)}{\lambda^{2}(x)}dx\right)}^{1/2}{\left(\int_{0}^{\infty }\;h(x){\left[\frac{{\bar{H}}_{k|n:G}(x)-{\bar{H}}_{k|n:G}^{\alpha }(x)}{\bar{H}(x)}\right]}^{2}dx\right)}^{1/2}\\ & ={\left(E\left(\frac{1}{\lambda^{2}(X)}\right)\right)}^{1/2}{\left(\int_{0}^{1}\;{\left[\frac{{\bar{G}}_{k|n:G}(u)-{\bar{G}}_{k|n:G}^{\alpha }(u)}{(1-u{)}^{2}}\right]}^{2}du\right)}^{1/2}, \end{array}$$

where the last equality follows from the change of variable u=H(x), giving the result. When 0<α<1, the inequality is reversed, and thus the theorem follows. □

In the final step, an upper bound for the CRTE is derived using the second moment $E\left(X^{2}\right)$, assuming that this moment exists. We now derive an alternative upper bound based on the second moment.

**Theorem** **10.** *Under the conditions of Theorem 8 such that* $E\left(X^{2}\right)<\infty$*, for* 2k≥n*, the following inequality holds when* α>1*,*

$$\xi_{\alpha }\left(T_{k|n:G}\right)\leq \frac{1}{\alpha -1}\sqrt{\eta_{k,n,\alpha }E\left(X^{2}\right)},$$

*where* $\eta_{k,n,\alpha }=\int_{0}^{1}\;{\left[g_{k|n:G}(u)\left(1-\alpha {\bar{G}}_{k|n:G}^{\alpha -1}(u)\right)\right]}^{2}du.$ *Conversely, when* 0<α<1*, the inequality is reversed.*

**Proof.**  From (2) and utilizing integration by parts, the CRTE of $T_{r|n:G}$ can be derived as:

$$\begin{array}{ll} \xi_{\alpha }\left(T_{k|n:G}\right) & =\frac{1}{\alpha -1}\int_{0}^{\infty }\;xh_{k|n:G}(x)\left(1-\alpha {\bar{H}}_{k|n:G}^{\alpha -1}(x)\right)dx \end{array},for\;\alpha \in \Omega .$$

If α>1, applying Cauchy–Schwarz inequality yields:

$$\begin{array}{ll} \xi_{\alpha }\left(T_{k|n:G}\right) & =\frac{1}{\alpha -1}\int_{0}^{\infty }\;x\sqrt{h(x)}\sqrt{h(x)}g_{k|n:G}(H(x))\left(1-\alpha {\bar{G}}_{k|n:G}^{\alpha -1}(H(x))\right)dx\\ & \leq \frac{1}{\alpha -1}{\left(\int_{0}^{\infty }\;x^{2}h(x)dx\right)}^{\frac{1}{2}}{\left(\int_{0}^{\infty }\;h(x){\left[g_{k|n:G}(H(x))\left(1-\alpha {\bar{H}}_{k|n:G}^{\alpha -1}(x)\right)\right]}^{2}dx\right)}^{\frac{1}{2}}\\ & ={\left(E\left(X^{2}\right)\right)}^{\frac{1}{2}}{\left(\int_{0}^{1}\;{\left[g_{k|n:G}(u)\left(1-\alpha {\bar{G}}_{k|n:G}^{\alpha -1}(u)\right)\right]}^{2}du\right)}^{\frac{1}{2}}, \end{array}$$

where the last equality follows from the change of variable u=H(x). When 0<α<1, the inequality is reversed, and thus the theorem follows. □

To illustrate the applicability of Theorems 8–10, we now present a representative example.

**Example** **4.** *Consider a linear consecutive 2-out-of-3:G system with lifetime*

$$T_{2|3:G}=max\left(min\left(X_{1},X_{2}\right),min\left(X_{2},X_{3}\right)\right),$$

*where the component lifetimes* $X_{i}$ *are i.i.d. with an exponential distribution with the cdf* $\bar{H}(x)=$ $e^{-\lambda x}$ *for* x>0*. The exponential distribution has a constant hazard rate,* $\lambda_{X}(x)=\lambda$*, so, it follows that* $E\left(\frac{1}{\lambda_{X}\left(T_{2|3:G,\alpha )}\right)}\right)=\frac{1}{\lambda }$*. Applying Theorem 8 yields the following bounds on the CRTE of the system*

$$\frac{1}{2\lambda \alpha }\leq \xi_{\alpha }\left(T_{2|3:G}\right)\leq \frac{1}{\lambda \alpha },\;for\;\alpha \in \Omega .\;$$

*Conversely, by revisiting Theorem 9, we can derive the bounds for the CRTE of the system as follows:*

$$\xi_{\alpha }\left(T_{2|3:G}\right)=\left\{\begin{array}{ll} \leq \frac{\sqrt{\Omega_{2,3,\alpha }}}{\lambda (\alpha -1)}, & \alpha >1\\ \geq \frac{\sqrt{\Omega_{2,3,\alpha }}}{\lambda (\alpha -1)}, & 0<\alpha <1 \end{array},\right.$$

*where* $\Omega_{2,3,\alpha }=\int_{0}^{1}\;\frac{\left.{\bar{G}}_{2|3:G}(u)-{\bar{G}}_{2|3:G}^{\alpha }(u\right)}{1-u}du$*. Additionally, noting that* $E\left(X^{2}\right)=\frac{2}{\lambda^{2}}$*, and referring to Theorem 10, we establish the following inequality*

$$\xi_{\alpha }\left(T_{2|3:G}\right)=\left\{\begin{array}{ll} \leq \frac{1}{\lambda (\alpha -1)}\sqrt{2\eta_{2,3,\alpha }}, & \alpha >1\\ \geq \frac{1}{\lambda (\alpha -1)}\sqrt{2\eta_{2,3,\alpha }}, & 0<\alpha <1, \end{array}\right.$$

*where* $\eta_{2,3,\alpha }=\int_{0}^{1}\;{\left[g_{2|3:G}(u)\left(1-\alpha {\bar{G}}_{2|3:G}^{\alpha -1}(u)\right)\right]}^{2}du$*.*

**Figure 3:**
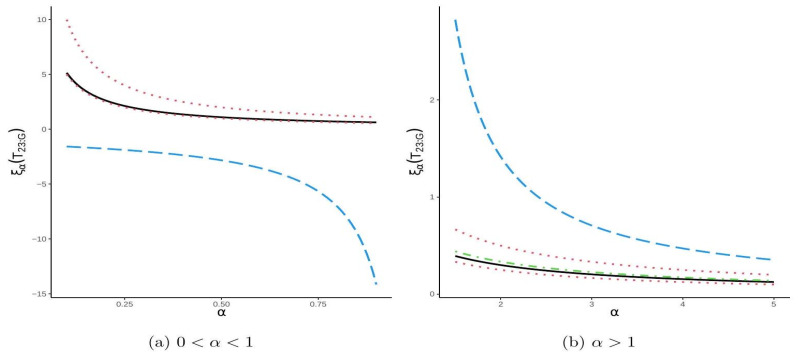
The plot of $\xi_{\alpha }\left(T_{2|3:G}\right)$ with respect to α as demonstrated in Example 4.

To illustrate the theoretical results, we compute both the exact values of the CRTE and the corresponding bounds established in Theorems 8–10. In the graphical representation, the exact values are plotted as a solid line, the bounds from Theorem 8 appear as a dotted line, those from Theorem 9 as a dashed line, and those from Theorem 10 as a dash-dotted line. This visualization facilitates the analysis of the relationship between $\xi_{\alpha }\left(T_{2|3:G}\right)$ and the entropy parameter α. The numerical results, shown in Figure 3, highlight this relationship and indicate that the upper bound derived in Theorem 9 provides the closest approximation to the exact values.

## 3. CRTE-Based Characterization of Consecutive k-out-of-n:G Systems

This section presents characterization results based on the CRTE properties of consecutive k-out-of-n:G systems. To this aim, we analyze a linear consecutive (n−i)-out-of-n:G system under the condition n≥2i, where i=0,1,…,n/2. A sequence of functions $f_{n}(x)$ is said to be complete in L(0,1) if, for every function g∈L(0,1), the condition

$$\int_{0}^{1}\;g\left(x\right)f_{n}\left(x\right)dx=0,\;for\;any\;n=1,2,\ldots ,$$

implies

g(x)=0 a.e. on (0,1).

We now present a lemma that follows directly from the Stone–Weierstrass Theorem (see Aliprantis and Burkinshaw [32]).

**Lemma** **1.** *For any increasing sequence of positive integers* $\left\{n_{j},j\geq 1\right\}$*, the sequence of polynomials* $\left\{x^{n_{j}}\right\}$ *is complete on* L(0,1) *if and only if for all* α∈Ω*,*

(13) $$\sum_{j=1}^{\infty }\;\;n_{j}^{-1}=\infty ,0<n_{1}<n_{2}<\cdots$$

*Hwang and Lin [33] provided an extension of the Müntz–Szász theorem, which will be employed in establishing the main results of this section.*

**Lemma** **2.** *Let* h(x) *be an absolutely continuous function on* (0,1) *with* f(0)f(1)≥0*, and suppose its derivative satisfies* $f^{\prime }(x)\neq 0$ *lmost everywhere on* (0,1)*. Then, assuming (13), the sequence* $\left\{h^{n_{j}}(x),j\geq 1\right\}$ *is complete on* L(0,1) *if and only if* h(x)*is monotone on*(0,1).

This lemma paves the way to establish a unique characterization of the parent distribution of a lifetime random variable through the CRTE of $T_{n-i|n:G}$.

**Theorem** **11.** *Let* $T_{n-i|n:G}^{X}\in C_{X}$ *and* $T_{n-i|n:G}^{Y}\in C_{Y}$*. Then* $H_{X}$ *and* $H_{Y}$ *belong to the same family of distributions, but for a change in location, if and only if for a fixed* k*,*

$$\xi_{\alpha }\left(T_{n-i|n:G}^{X}\right)=\xi_{\alpha }\left(T_{n-i|n:G}^{Y}\right),\mathrm{when}\;0\leq i\leq n/2.$$

**Proof.**  For the necessity part, since $H_{X}$ and $H_{Y}$ belong to the same family of distributions, but for a change in location, then ${\bar{H}}_{Y}(y)={\bar{H}}_{X}(y-c)$, for all y≥c and c∈R. Then, it is clear that

$$\begin{array}{ll} \xi_{\alpha }\left(T_{n-i|n:G}^{Y}\right) & =\frac{1}{\alpha -1}\int_{c}^{\infty }\;\left[{\bar{H}}_{n-i|n:G}^{Y}(y)-{\bar{H}}_{n-i|n:G}^{\alpha ,Y}(y)\right]dy\\ & =\frac{1}{\alpha -1}\int_{c}^{\infty }\;\left[{\bar{H}}_{n-i|n:G}^{X}(y-c)-{\bar{H}}_{n-i|n:G}^{\alpha ,X}(y-c)\right]dy\\ & =\frac{1}{\alpha -1}\int_{0}^{\infty }\;\left[{\bar{H}}_{n-i|n:G}^{X}(x)-{\bar{H}}_{n-i|n:G}^{\alpha ,X}(x)\right]dx=\xi_{\alpha }\left(T_{n-i|n:G}^{X}\right), \end{array}$$

where the last equality is obtained by the change of x=y−c. To establish the sufficiency part, we first note that for a consecutive (n−i)-out-of-n:G system, the following equation holds:

$${\bar{G}}_{n-i|n:G}(u)=\left(i+1\right){\left(1-u\right)}^{n-i}-i{\left(1-u\right)}^{n-i+1},0<u<1,$$

where n≥2i and i ranges from 1 to n/2. Employing this, relation (8) can be rewritten as follows:

(14) $$\xi_{\alpha }\left(T_{n-i|n:G}^{X}\right)=\int_{0}^{1}\;\frac{\chi_{\alpha }\left({\bar{G}}_{n-i|n:G}(u)\right)}{h\left(H^{-1}(u)\right)}du,$$

for i=1,2,…,n. The same argument also holds for Y. Given the assumption that $\xi_{\alpha }\left(T_{n-i|n:G}^{X}\right)=$ $\xi_{\alpha }\left(T_{n-i|n:G}^{Y}\right)$, using relation (14), we can write

(15) $$\int_{0}^{1}\;(1-u{)}^{n}\phi_{i,n,\alpha }(1-u)\left(h_{X}\left(H_{X}^{-1}\left(u\right)\right)-h_{Y}\left(H_{Y}^{-1}\left(u\right)\right)\right)du=0,$$

where

$$\begin{array}{c} \phi_{i,n,\alpha }(1-u)=(i+1)(1-u{)}^{-i}-i(1-u{)}^{-i+1}-(1-u{)}^{n\alpha -1}{\left((i+1)(1-u{)}^{-i}-i(1-u{)}^{-i+1}\right)}^{\alpha },\\ \mathrm{for}\;\mathrm{all}\;0<u<1\mathrm{and}\;\mathrm{for}\;\mathrm{all}\;\alpha \in \Omega . \end{array}$$

By taking z=1−u, Equation (15) can be rewritten as follows:

$$\int_{0}^{1}\;z^{n}\phi_{i,n,\alpha }(1-z)\left(h_{X}\left(H_{X}^{-1}(1-z)\right)-h_{Y}\left(H_{Y}^{-1}(1-z)\right)\right)du=0.$$

By invoking Lemma 2 and defining the function

$$\psi (z)=\phi_{i,n,\alpha }(1-z)\left(h_{X}\left(H_{X}^{-1}(1-z)\right)-h_{Y}\left(H_{Y}^{-1}(1-z)\right)\right),$$

and analyzing the complete sequence $\left\{z^{n},n\geq 1\right\}$, we arrive at the conclusion that

$$h_{X}\left(H_{X}^{-1}(1-z)\right)=h_{Y}\left(H_{Y}^{-1}(1-z),a.e.\;z\in (0,1),\right.$$

or equivalently $h_{X}\left(H_{X}^{-1}(x)\right)=h_{Y}\left(H_{Y}^{-1}(x)\right)$ for all x∈(0,1). It follows that $H_{X}^{-1}(x)=H_{Y}^{-1}(x)+d$ for a constant d. This means that X and Y have the same distribution functions, but for a location change, thus completing the proof. □

Since a consecutive n-out-of-n:G system reduces to a classical series system, the following corollary provides a characterization of its CRTE.

**Corollary** **2.** *Let* $T_{n|n:G}^{X}\in C_{X}$ *and* $T_{n|n:G}^{Y}\in C_{Y}$*. Then* $H_{X}$ *and* $H_{Y}$ *belong to the same family of distributions, but for a change in location, if and only if*

$$\xi_{\alpha }\left(T_{n|n:G}^{X}\right)=\xi_{\alpha }\left(T_{n|n:G}^{Y}\right),\mathrm{for}\;\mathrm{all}\;n\geq 1.$$

Another helpful characterization is provided in the following theorem.

**Theorem** **12.** *Under the conditions of Theorem 11,* $H_{X}$ *and* $H_{Y}$ *belong to the same family of distributions, but for a change in location and scale, if and only if for a fixed* k*,*

(16) $$\frac{\xi_{\alpha }\left(T_{k|n:G}^{X}\right)}{\xi_{\alpha }(X)}=\frac{\xi_{\alpha }\left(T_{k|n:G}^{Y}\right)}{\xi_{\alpha }(Y)},\;\frac{n}{2}\leq k\leq n.$$

**Proof.**  The necessity is straightforward, so we must now establish the sufficiency aspect. Leveraging Equations (3) and (14), we can derive

(17) $$\left(\frac{\xi_{\alpha }\left(T_{i|n:G}^{X}\right)}{\xi_{\alpha }(X)}\right)=\int_{0}^{1}\;\chi_{\alpha }\left({\bar{G}}_{i|n:G}(u)\right)\frac{h_{X}\left(H_{X}^{-1}(u)\right)}{\xi_{\alpha }(X)}du.$$

An analogous argument can be made for $\xi_{\alpha }\left(T_{k|n:G}^{Y}\right)/\xi_{\alpha }(Y)$. If relation (16) holds for two cdfs $H_{X}$ and $H_{Y}$, then we can infer from Equation (17) that

$$\int_{0}^{1}\;\chi_{\alpha }\left({\bar{G}}_{i|n:G}(u)\right)\frac{h_{X}\left(H_{X}^{-1}(u)\right)}{\xi_{\alpha }(X)}du=\int_{0}^{1}\;\chi_{\alpha }\left({\bar{G}}_{i|n:G}(u)\right)\frac{h_{Y}\left(H_{Y}^{-1}(u)\right)}{\xi_{\alpha }(Y)}du.$$

Let us set $c=\xi_{\alpha }(Y)/\xi_{\alpha }(X)$. Using similar arguments as in the proof of Theorem 11, we can write

$$\int_{0}^{1}\;z^{n}\phi_{i,n,\alpha }(1-z)\left(ch_{X}\left(H_{X}^{-1}(1-z)\right)-h_{Y}\left(H_{Y}^{-1}(1-z)\right)\right)du=0.$$

The proof is then completed by using similar arguments to those in Theorem 11.

Applying Theorem 12, we obtain the following corollary. □

**Corollary** **3.** *Suppose the assumptions of Corollary 2,* $H_{X}$ *and* $H_{Y}$*, belong to the same family of distributions, but for a change in location and scale, if and only if*

$$\frac{\xi_{\alpha }\left(T_{n|n:G}^{X}\right)}{\xi_{\alpha }(X)}=\frac{\xi_{\alpha }\left(T_{n|n:G}^{Y}\right)}{\xi_{\alpha }(Y)},\mathrm{for}\;\mathrm{all}\;n\geq 1.$$

The following theorem provides a characterization of the exponential distribution based on CRTE in the context of consecutive k-out-of-n:G systems. This result not only offers theoretical insight but also forms the basis for a novel goodness-of-fit test that can be applied to empirical data to assess conformity to the exponential model. To derive this result, we begin by introducing the lower incomplete beta function, defined as

$$B(t;a,b)=\int_{0}^{t}\;x^{a-1}(1-x{)}^{b-1},0<t<1,$$

where a and b are positive real numbers. If t=0, it simplifies to the complete beta function. We now proceed to state the main theorem.

**Theorem** **13.** *Let* $T_{k|n:G}\in C_{X}$*. Then* X *has an exponential distribution with mean* λ *if and only if for a fixed* k*,*

(18) $$\xi_{\alpha }\left(T_{k|n:G}\right)=\frac{\alpha \xi_{\alpha }(X)}{\alpha -1}\left[\frac{n+1}{k(k+1)}-g\right],$$

*where* $g=\frac{(n-k+1{)}^{\alpha (k+1)}}{(n-k{)}^{\alpha k}}B\left(\frac{n-k}{n-k+1};\alpha (k-1),\alpha +1\right),\;$ *when* n/2≤k≤n.

**Proof.**  For an exponentially distributed random variable X with mean λ, calculated directly using (3), we have $\xi_{\alpha }(X)=\frac{\lambda }{\alpha }$. Additionally, since $h\left(H^{-1}(u)\right)=\frac{\left(1-u\right)}{\lambda }$, applying Equation (8) results in:

$$\begin{array}{ll} \xi_{\alpha }\left(T_{k|n:G}\right) & =\int_{0}^{1}\;\frac{\chi_{\alpha }\left({\bar{G}}_{k|n:G}(u)\right)}{h\left(H^{-1}(u)\right)}du=\frac{\lambda \alpha }{\alpha }\int_{0}^{1}\;\frac{\chi_{\alpha }\left({\bar{G}}_{k|n:G}(u)\right)}{1-u}du\\ & =\alpha \xi_{\alpha }(X)\int_{0}^{1}\;\frac{\chi_{\alpha }\left({\bar{G}}_{k|n:G}(u)\right)}{1-u}\;\mathrm{du}. \end{array}$$

To derive the second term, we first observe that by recalling (5), we obtain

(19) $$\int_{0}^{1}\;\frac{{\bar{G}}_{k|n:G}(u)}{1-u}du=\frac{n+1}{k(k+1)}.$$

Setting A=(n−k+1) and B=(n−k), upon recalling (5), it holds that

$$\begin{array}{ll} \int_{0}^{1}\;\frac{{\bar{G}}_{k|n:G}^{\alpha }(u)}{1-u}du & =\int_{0}^{1}\;(1-u{)}^{\alpha k-1}(A-B(1-u){)}^{\alpha }du\\ & =A^{\alpha }\int_{0}^{1}\;z^{\alpha k-1}{\left(1-\frac{B}{A}z\right)}^{\alpha }dz,(\mathrm{taking}\;z=1-u)\\ & =\frac{A^{\alpha \left(k+1\right)}}{B^{\alpha k}}\int_{0}^{\frac{B}{A}}\;w^{\alpha k-1}(1-w{)}^{\alpha }dw,\left(\mathrm{taking}\;w=\frac{B}{A}z\right) \end{array}$$

(20) $$=\frac{A^{\alpha \left(k+1\right)}}{B^{\alpha k}}B\left(\frac{n-k}{n-k+1};\alpha (k-1),\alpha +1\right).\;\;$$

By combining the results from Equations (19) and (20), we derive the following relationship:

(21) $$\;\int_{0}^{1}\;\frac{\chi_{\alpha }\left({\bar{G}}_{k|n:G}(u)\right)}{1-u}du=\frac{1}{\alpha -1}\left[\frac{n+1}{k(k+1)}-E\right],$$

$E=\frac{A^{\alpha (k+1)}}{B^{\alpha r}}B\left(\frac{n-k}{n-k+1};\alpha (k-1),\alpha +1\right),\;$ where the necessity is derived. To prove the sufficiency condition, we begin by assuming that Equation (18) is satisfied for a specific value of k. Building on the methodology outlined in the proof of Theorem 11 and incorporating the key result from Equation (21), we establish the following relationship

$$\int_{0}^{1}\;\frac{\chi_{\alpha }\left({\bar{G}}_{k|n:G}(u)\right)}{h\left(H^{-1}(u)\right)}du=\alpha \xi_{\alpha }\left(X\right)\int_{0}^{1}\;\frac{\chi_{\alpha }\left({\bar{G}}_{k|n:G}\left(u\right)\right)}{1-u}du,$$

which is equivalent to

$$\int_{0}^{1}\;z^{n}\phi_{i,n,\alpha }(1-z)\left[\frac{1}{h\left(H^{-1}\left(1-z\right)\right)}-\frac{\alpha \xi_{\alpha }\left(X\right)}{z}\right]du=0,$$

using similar arguments to those in Theorem 11. Applying Lemma 2 to the function

$$\psi \left(x\right)=\phi_{i,n}\left(1-z\right)\left[\frac{1}{h\left(H^{-1}\left(1-z\right)\right)}-\frac{\alpha \xi_{\alpha }\left(X\right)}{z}\right],$$

and utilizing the complete sequence $\left\{z^{n},n\geq 1\right\}$, we can deduce that

$$\frac{1}{h\left(H^{-1}(1-z)\right)}=\frac{\alpha \xi_{\alpha }(X)}{z},\;a.e.\;z\in \left(0,1\right),$$

which is equivalent to

$$\frac{1}{h\left(H^{-1}(w)\right)}=\frac{\alpha \xi_{\alpha }(X)}{1-w},\;a.e.\;w\in \left(0,1\right),$$

by taking w=1−z. This implies that

$$\frac{dH^{-1}(w)}{dw}=\frac{\alpha \xi_{\alpha }(X)}{\left(1-w\right)},$$

since $\frac{dH^{-1}(w)}{dw}=\frac{1}{h\left(H^{-1}(w)\right)}$. Integrating both sides of the above relation, we obtain

$$\int dH^{-1}(w)=\int \frac{\alpha \xi_{\alpha }(X)}{\left(1-w\right)}dw,$$

which implies $H^{-1}(w)=-\alpha \xi_{\alpha }(X)log(1-$ w)+d, where d is a constant. Since $H^{-1}\left(0\right)=0$ and the logarithm function is defined on (0,∞), we find that d=0. Substituting $x=H^{-1}\left(w\right),$ we obtain $x=-\alpha \xi_{\alpha }(X)log\;(1-H(x))$ leading to $H(x)=1-e^{-\alpha \xi_{\alpha }(X)x}$ for x>0. The condition x>0 arises from the domain of logarithm function (0,∞), implying 0<w<1, and thus 0<x<∞, since $0<H^{-1}\left(w\right)<\infty$ by noting that $H^{-1}\left(1\right)=\infty .$ This confirms that $X∼E\left(\alpha \xi_{\alpha }(X)\right)$ and thereby establishes the theorem. □

## 4. Nonparametric Testing of Dispersive Ordering via CRTE

We propose a simple nonparametric procedure for testing dispersive ordering in the two-sample setting. Let $X_{1},X_{2},\ldots ,X_{N}$ and $Y_{1},Y_{2},\ldots ,Y_{M}$ be independent random samples drawn from distributions X and Y, respectively. Note that $X\leq_{\mathrm{disp}}Y$ and $Y\leq_{\mathrm{disp}}X$ if and only if F(x)= G(x+c) for some real c and all real x. In this case, we say that $X{=}_{disp}Y$. Our goal is to test the null hypothesis $H_{0}:X{=}_{disp}Y$ against the alternative $H_{1}:X\leq_{\mathrm{disp}}Y$, and $X\neq_{disp}Y$, by using the functional

$$\Delta_{k,n,\alpha }=\xi_{\alpha }\left(T_{k|n:G}^{X}\right)-\xi_{\alpha }\left(T_{k|n:G}^{Y}\right),$$

as a discrepancy measure from $H_{0}$ in favor of $H_{1}$ due to Theorem 4. A natural idea is to estimate $\Delta_{k,n,\alpha }$ and reject $H_{0}$ whenever the estimate is large enough. To estimate $\Delta_{k,n,\alpha }$, we simply replace both $\xi_{\alpha }\left(T_{k|n:G}^{X}\right)$ and $\xi_{\alpha }\left(T_{k|n:G}^{Y}\right)$ by their natural estimators ${\hat{\xi }}_{N,\alpha }\left(T_{k|n:G}^{X}\right)$ and ${\hat{\xi }}_{M,\alpha }\left(T_{k|n:G}^{Y}\right)$ respectively. Hence, it turns out that the estimation of $\Delta_{k,n,\alpha }$ is given by

$${\hat{\Delta }}_{k,n,\alpha }={\hat{\xi }}_{N,\alpha }\left(T_{k|n:G}^{X}\right)-{\hat{\xi }}_{M,\alpha }\left(T_{k|n:G}^{Y}\right).$$

Let us assume a sequence of i.i.d. continuous, non-negative random variables $X_{1},X_{2},\ldots ,X_{N}$, where $X_{1:N}\leq X_{2:N}\leq \cdots \leq X_{N:N}$ denote their order statistics. We denote the empirical distribution function corresponding to H(x) of the sample by

$${\hat{H}}_{N}(x)=\frac{1}{N}\sum_{i=1}^{N}\;I_{\left(X_{i}\leq x\right)}=\left\{\begin{array}{l} 0,x<x_{1:N}\\ \frac{i}{N},x_{i:N}\leq x\leq x_{i+1:N},\left(i=1,2,\ldots ,N-1\right),\\ 1,x>x_{N:N} \end{array}\right.$$

where $I_{A}$ is the indicator function of A, that is, $I_{A}=1$ if A is true. A nonparametric estimator of the CRTE for a consecutive k-out-of-n:G system, based on the L-functional estimator, is given by:

$${\hat{\xi }}_{N,\alpha }\left(T_{k|n:G}^{X}\right)=\int_{0}^{\infty }\;xJ_{k,n,\alpha }\left({\hat{H}}_{N}(x)\right)d{\hat{H}}_{N}(x)=\frac{1}{N}\sum_{i=1}^{N}\;J_{k,n,\alpha }\left(\frac{i}{N}\right)X_{\left(i\right)},\;\mathrm{for}\;\mathrm{all}\;\alpha >0,$$

where

$$J_{k,n,\alpha }(u)=\frac{g_{k}(u)}{\alpha -1}\left(1-\alpha {\bar{G}}_{k,n}^{\alpha -1}(u)\right),0<u<1,$$

and ${\bar{G}}_{k|n:G}(u)$ is defined in (5), with $g_{k|n:G}(u)=-\frac{d{\bar{G}}_{k|n:G}(u)}{du}$. The next theorem establishes the asymptotic normality of the estimator (3). First, we need the following lemma.

**Lemma** **3.** *For* 2k≥n*, where* n *is fixed, and* α>0*, we have*

$$\left|J_{k,n,\alpha }(u)\right|\leq \frac{k\left(n-r+1\right)}{\left|\alpha -1\right|},\;for\;0\leq u\leq 1.$$

**Proof.**  Let n be fixed. Since $\alpha {\bar{G}}_{k,n}^{\alpha -1}(u)\geq 0$, for all α≥0, it follows that $1-\alpha {\bar{G}}_{k,n}^{\alpha -1}(u)\leq 1$, for 0<u<1. Additionally, since k(n−k+1)−(k+1)(n−k)(1−u)≤k(n−k+1),0<u<1, we obtain

$$g_{k|n:G}(u)\leq k(n-k+1),0\leq u\leq 1.$$

Consequently, for α>0, the following inequality holds:

$$\begin{array}{ll} \left|J_{k,n,\alpha }(u)\right| & =\left|\frac{g_{k|n:G}(u)}{\alpha -1}\left(1-\alpha {\bar{G}}_{k,n}^{\alpha -1}(u)\right)\right|\\ & \leq \left|\frac{g_{k|n:G}\left(u\right)}{\alpha -1}\right|\leq \frac{k\left(n-k+1\right)}{\left|\alpha -1\right|}, \end{array}$$

which completes the proof. □

Similar arguments hold for the ${\hat{\xi }}_{M,\alpha }\left(T_{k|n:G}^{Y}\right)$. The subsequent theorem establishes the asymptotic normality of the test statistic ${\hat{\Delta }}_{k,n,\alpha }$.

**Theorem** **14.** *Assume that* $E\left(X^{2}\right)<\infty$ *and* $E\left(Y^{2}\right)<\infty$ *such that* $\sigma_{k,n,\alpha }^{2}\left(H_{X}\right)>0$*, and* $\sigma_{k,n,\alpha }^{2}\left(H_{Y}\right)>0$*. Let* S=M+N *and suppose that for some* 0<τ<1*, we have*

$$\frac{N}{S}\to \tau ,\;\frac{M}{S}\to 1-\tau \;\;\mathrm{as}\;\;min\left\{N,M\right\}\to \infty .$$

*For* 2r≥n*, as* min{N,M}→∞*, then* $\sqrt{S}\left({\hat{\Delta }}_{k,n,\alpha }-\Delta_{k,n,\alpha }\right)$ *is normal with mean zero and the finite variance*

$$\sigma_{k,n,\alpha }^{2}\left(H_{X},H_{Y}\right)=\frac{\sigma_{k,n,\alpha }^{2}\left(H_{X}\right)}{\tau }+\frac{\sigma_{k,n,\alpha }^{2}\left(H_{Y}\right)}{1-\tau },$$

*where*

(22) $${\;\;\sigma }_{k,n,\alpha }^{2}\left(H_{X}\right)=\int_{0}^{\infty }\;\int_{0}^{\infty }\;\left(H_{X}\left(\min\left(x,y\right)\right)-H_{X}\left(x\right)H_{X}\left(y\right)\right)J_{k,n,\alpha }\left(x\right)J_{k,n,\alpha }\left(y\right)dx\;dy,$$

*and* $\sigma_{k,n,\alpha }^{2}\left(H_{Y}\right)$ *is defined similarly for* α>0.

**Proof.**  The smooth function $J_{2}$ is bounded (by Lemma 3) and continuous. Therefore, applying Theorems 2 and 3 of Stigler [34], it follows that $\sqrt{N}\left({\hat{\xi }}_{\alpha }\left(T_{k|n:G}\right)-\xi_{\alpha }\left(T_{k|n:G}\right)\right)$ converges in distribution to a normal distribution with mean zero and finite variance $\sigma_{k,n,\alpha }^{2}\left(H_{X}\right)>0$ as N goes infinity. This conclusion arises from the property that convergence in distribution is, in general, preserved under convolution. □

Since expression (22) depends on the unknown distribution function, a consistent estimator is required. This can be obtained using the representation introduced by Jones and Zitikis [35], given as follows:

$${\hat{\sigma }}_{k,n,\alpha }^{2}\left(H_{X}\right)=\sum_{i=1}^{N-1}\;\sum_{j=1}^{N-1}\;\left(min\left(\frac{i}{N},\frac{j}{N}\right)-\frac{i}{N}\frac{j}{N}\right)J_{k,n,\alpha }\left(\frac{i}{N}\right)J_{k,n,\alpha }\left(\frac{j}{N}\right)\left(X_{i+1:N}-X_{i:N}\right)\left(X_{j+1:N}-X_{j:N}\right),$$

and ${\hat{\sigma }}_{k,n,\alpha }^{2}\left(H_{X}\right)$ is given similarly for α>0. The decision rule at significance level q is to reject $H_{0}$ in favour of $H_{1}$ if

$$\left|\frac{{\hat{\Delta }}_{k,n,\alpha }}{\sqrt{\frac{{\hat{\sigma }}_{k,n,\alpha }^{2}\left(H_{X}\right)}{N}+\frac{{\hat{\sigma }}_{k,n,\alpha }^{2}\left(H_{Y}\right)}{M}}}\right|>z_{1-q},$$

where $z_{1-q}$ is the (1−q)-quantile of the standard normal distribution. In the following section, we employ Monte Carlo simulation to evaluate and compare the statistical power of our proposed test statistic against several alternative statistics. This analysis focuses on assessing their performance in fitting the exponential distribution to randomly sampled data.

### 4.1. Monte Carlo Evaluation of the CRTE-Based Test

To assess the performance of the proposed test, we conducted a comparative simulation study focusing on empirical power. Specifically, we evaluated the performance of the test statistic ${\hat{\Delta }}_{k,n,\alpha }$ against three recent tests for dispersive ordering: the test proposed by Aly [36], denoted by $t_{N}$; the test by Marzec and Marzec [37], denoted by $L_{0.5,N}$; and the test introduced by Sordo et al. [38], denoted by $S_{N}$. Notably, $\Lambda_{N}$ is based on Gini’s mean difference and has been evaluated under various estimators for its asymptotic variance. The simulation encompassed several scenarios, and we compared the empirical powers of all test statistics across these settings.

*Exponential Distribution*: For this scenario, X∼Exp(1) and Y∼Exp(1/β) where β is varied from 1 to 2. The null hypothesis is then represented by the case where β=1.

*Pareto Distribution*: For this scenario, the random variable X∼Pa(10,3) and Y∼ Pa(10/β,3) where β varied from 1 to 2. The null hypothesis is then represented by the case where β=1.

*Gamma Distribution*: For this scenario, X∼G(2,1) and Y∼G(β,1) where β varied from 2 to 3. The null hypothesis is then represented by the case where β=2.

Weibull Distribution: For this scenario, X∼W(2,1) and Y∼G(β,1) where β varied from 1 to 2. The null hypothesis is then represented by the case where β=2.

**Figure 4:**
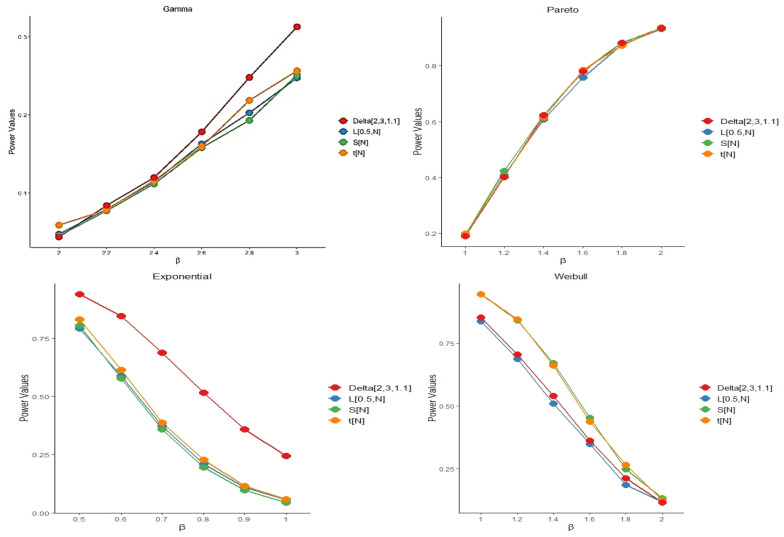
The plot of power comparisons of the tests in significance level α=0.05 when *n* = 25.

**Figure 5:**
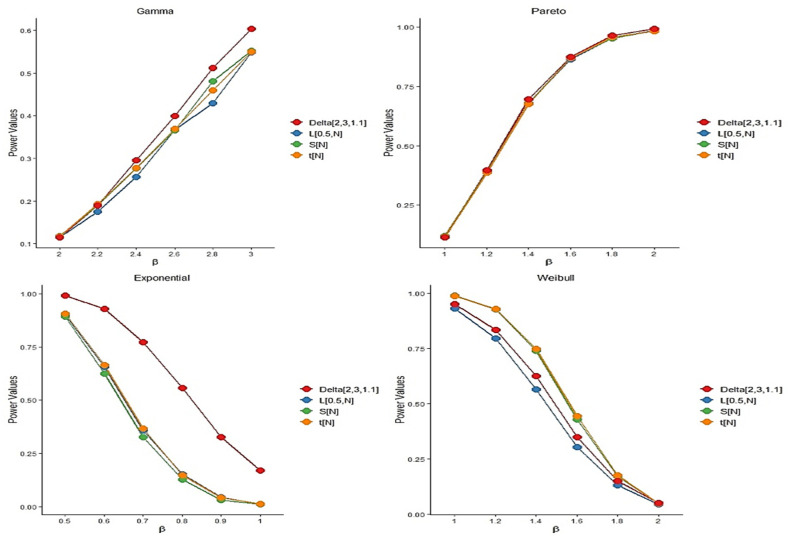
The plot of power comparisons of the tests in significance level α=0.05 when *n* = 50.

**Figure 6:**
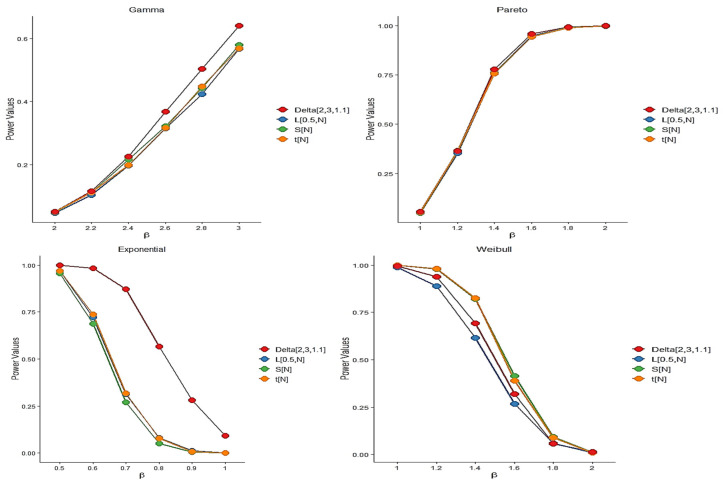
The plot of power comparisons of the tests in significance level α=0.05 when *n* = 100.

Different choices of k,n, and α allow for the construction of various versions of the proposed test statistic. Based on our simulation results, a suitable value for the tuning parameter α\alphaα is approximately 1.1, with n=3 and k=2. By “suitable,” we refer to a choice that maintains the nominal significance level under the null hypothesis while yielding high empirical power under the alternative. The selected parameter values for each model are reported alongside the corresponding results. For each scenario, we computed the empirical power of the test statistics using 5000 independently generated sample pairs with sizes n=m=25,50, and 100. To ensure reproducibility and facilitate open scientific practice, all analyses were performed using R version 4.4.1. Power was measured as the proportion of simulations in which the test statistic exceeded its corresponding critical value. The simulation outcomes are summarized in Figure 4, Figure 5 and Figure 6. As expected, the empirical power of all tests increases with the sample size, confirming their consistency. While the test statistic $Z_{2,3,2}$ performs comparably to the existing methods, it shows particularly strong performance at larger sample sizes. The probability distributions used in our study are presented in Table 2, along with their shape and scale parameters and the empirical power comparisons of the proposed test and existing methods at significance level α = 0.05 are shown in Table 3. In addition, further power comparisons for Gamma and Pareto distributions at significance level α = 0.05 are summarized in Table 4.

### 4.2. Real Data Application of the CRTE-Based Test

To illustrate the practical applicability of the proposed test statistics for validating dispersive order, we present a numerical example based on real-world data. Specifically, we apply the four test statistics to compare survival times from two groups of male RFM strain mice, originally reported by Hoel [39] as given in Table 5. The first group, with survival times denoted by X, was kept under conventional laboratory conditions and died from thymic lymphoma. The second group, denoted by Y, was maintained in a germ-free environment and succumbed to the same cause. The objective is to formally assess whether the dispersive ordering hypothesis holds between the two survival distributions. The statistical test results for the real dataset are summarized in Table 6, showing the *p*-values for the different test statistics.

These results consistently support the initial graphical indication, confirming the hypothesis that $X\leq_{\mathrm{disp}}Y$.

For the second case study, we analyze two sets of life test data for distinct snubber designs in a toaster component, sourced from Table 8.3.1 in Nelson [40], as presented in Table 7. Specifically, the data represent life test results for two snubber designs in a toaster component. The measurements indicate the number of toaster cycles (operations) until the snubber fails, causing the toaster to pop and eject the toast. The results of the statistical tests for the second real dataset are presented in Table 8, providing evidence on the hypothesis evaluation.

The results indicate that there is no evidence to reject the null hypothesis, supporting the assertion that $H_{0}:X{=}_{disp}Y$. Additional numerical results can be found in the Appendix A.

## 5. Conclusions and Future Directions

This paper has developed a comprehensive analysis of CRTE in the framework of linear consecutive k-out-of-n:G systems, providing new perspectives on both the quantification of uncertainty and the characterization of reliability structures. One key contribution was to establish a clear relationship between the CRTE of systems with general continuous lifetime distributions and their counterparts under uniform distributions. This result not only enriches the theoretical foundations of entropy-based reliability measures but also enables simplified computation in applied reliability contexts.

Given the challenges of deriving closed-form CRTE expressions in large or analytically complex systems, we proposed several upper and lower bounds that offer practical approximations when exact evaluation is infeasible. Beyond these structural findings, we introduced a nonparametric CRTE-based test for dispersive ordering, rigorously established its asymptotic distribution, and validated its performance through extensive Monte Carlo simulations. The test consistently demonstrated superior power and stability compared to existing alternatives. Furthermore, a real data study on survival times of RFM strain mice illustrated the interpretability and practical value of CRTE-based methods in empirical reliability analysis, underscoring their relevance for uncertainty quantification in practice.

In summary, this work advances both the theoretical development and the applied utility of CRTE in reliability engineering and information theory. It is worth noting that the CRTE measure is intentionally designed to be flexible and adaptable across different system sizes. Its formulation is scalable to large-scale systems such as satellite constellations without significantly increasing computational complexity. While the CRTE has demonstrated robust performance in capturing essential system characteristics, larger sizes may require extensive numerical computations. Nevertheless, our framework remains effective even as the scale increases, grounded in these fundamental principles. It positions CRTE as a versatile entropy-based tool for uncertainty quantification, reliability assessment, and statistical inference in complex systems. At the same time, several important directions remain open for further research: (i). Extending CRTE formulations to systems with dependent component lifetimes as investigated by Eryılmaz [8] allows for a more comprehensive analysis. (ii). Investigating CRTE in non-stationary or time-varying environments, where component failure rates evolve dynamically, to improve dynamic reliability assessment. (iii). Developing multivariate generalizations of CRTE that capture joint uncertainty in multi-component or multi-state systems. (iv). Designing efficient numerical algorithms and approximation schemes to enable CRTE analysis in high-dimensional or very large-scale systems. (v). Broadening the CRTE framework to include additional goodness-of-fit procedures and model selection criteria alongside dispersive-ordering tests. (vi). Performing systematic comparisons between CRTE and other entropy measures (e.g., cumulative Rényi entropy, fractional cumulative entropy) across diverse reliability settings to establish practical guidelines.

Addressing these challenges will further consolidate CRTE’s role as a probabilistic entropy measure for reliability modeling, while opening new opportunities for applications in uncertainty quantification, survival analysis, engineering system reliability, and complex system modeling.

## Figures and Tables

**Table 1:** The exact value and bounds for $\xi_{\alpha }(X)\left(T_{5|10:G}\right)$ for different choices of α.

α	$\xi_{\alpha }(X)\left(T_{5|10:G}\right)$	$L_{\alpha }\left(T_{5|10:G}\right)$	$B_{2,\alpha }$	$U_{\alpha }\left(T_{5|0:G}\right)$
0.1	1.978022	0.250797	1.876491	2.884636
0.2	1.731334	0.195623	1.820220	2.674001
0.5	1.318842	0.120960	1.697049	2.215572
0.8	1.097536	0.089033	1.611232	1.90664
1.2	0.915607	0.066600	1.527334	1.618696
2.0	0.708744	0.044955	1.414099	1.258151
2.5	0.628055	0.037566	1.363996	1.110122
3.0	0.566839	0.032342	1.323560	0.996017

**Table 2:** Probability distributions with the shape parameter β and the scale parameter γ.

Exponential	$f(x)=\frac{1}{\beta }e^{-\frac{x}{\beta }}$,	x>0,β>0
Weibull	$f(x)=\frac{\beta }{\gamma^{\beta }}x^{\beta -1}e^{-{\left(\frac{x}{\gamma }\right)}^{\beta }}$,	x>0,γ,β>0
Gamma	$f(x)=\frac{1}{\gamma^{\beta }\Gamma (\beta )}x^{\beta -1}e^{-\frac{x}{\gamma }}$	x>0,γ,β>0
Pareto	$f(x)=\frac{\beta \gamma^{\beta }}{(x+\gamma {)}^{\beta +1}}$,	x>0,γ,β>0

**Table 3:** Power comparisons of the tests in significance level α=0.05.

PDF Exponential Weibull										
N = M	γ	L_0.5_,N	S_n_	t_n_	$\hat{∆}$_2.3_,_1.1_	β	L_0.5_,N	S_n_	t_n_	$\hat{∆}$_2.3_,_1.1_
25	0.5	0.7924	0.8042	0.8314	0.9400	1.0	0.8362	0.9440	0.9438	0.8512
	0.6	0.5876	0.5782	0.6148	0.8454	1.2	0.6874	0.8402	0.8444	0.7042
	0.7	0.3730	0.3584	0.3882	0.6888	1.4	0.5098	0.6706	0.6602	0.5392
	0.8	0.2108	0.1946	0.2276	0.5160	1.6	0.3476	0.4522	0.4364	0.3612
	0.9	0.1104	0.0974	0.1158	0.3586	1.8	0.1848	0.2480	0.2648	0.2126
	1.0	0.0558	0.0440	0.0590	0.2440	2.0	0.1172	0.1318	0.1254	0.1156
50	0.5	0.9004	0.8922	0.9064	0.9912	1.0	0.9308	0.9898	0.9876	0.9508
	0.6	0.6566	0.6244	0.6644	0.9276	1.2	0.7958	0.9266	0.9294	0.8350
	0.7	0.3578	0.3272	0.3666	0.7712	1.4	0.5650	0.7388	0.7476	0.6256
	0.8	0.1514	0.1282	0.1484	0.5578	1.6	0.3044	0.4290	0.4430	0.3482
	0.9	0.0456	0.0314	0.0418	0.3264	1.8	0.1298	0.1720	0.1770	0.1496
	1.0	0.0110	0.0108	0.0134	0.1708	2.0	0.0444	0.0488	0.0482	0.0506
100	0.5	0.9710	0.9576	0.9702	0.9998	1.0	0.9888	0.9996	0.9992	0.9944
	0.6	0.7220	0.6866	0.7364	0.9842	1.2	0.8898	0.9802	0.9824	0.9378
	0.7	0.3124	0.2698	0.3180	0.8720	1.4	0.6150	0.8210	0.8262	0.6918
	0.8	0.0808	0.0502	0.0780	0.5668	1.6	0.2678	0.4146	0.3886	0.3192
	0.9	0.0118	0.0058	0.0078	0.2814	1.8	0.0596	0.0932	0.0886	0.0572
	1.0	0.0014	0.0002	0.0014	0.0924	2.0	0.0092	0.0106	0.0098	0.0136

**Table 4:** Power comparisons of the tests for Gamma and Pareto distributions at significance level *α* = 0.05.

PDF		Gamma				Pareto				
N = M	β	L_0.5_,N	S_n_	t_n_	$\hat{∆}$_2.3_,_1.1_	β	L_0.5_,N	S_n_	t_n_	$\hat{∆}$_2.3_,_1.1_
25	2.0	0.0476	0.0454	0.0592	0.0440	1.0	0.1906	0.1930	0.1972	0.1894
	2.2	0.0796	0.0774	0.0788	0.0844	1.2	0.4074	0.4236	0.4024	0.4010
	2.4	0.1146	0.1120	0.1164	0.1202	1.4	0.6074	0.6234	0.6144	0.6234
	2.6	0.1632	0.1582	0.1598	0.1784	1.6	0.7576	0.7842	0.7852	0.7810
	2.8	0.2030	0.1932	0.2188	0.2482	1.8	0.8760	0.8834	0.8730	0.8828
	3.0	0.2476	0.2514	0.2568	0.3130	2.0	0.9324	0.9338	0.9374	0.9354
50	2.0	0.1148	0.1186	0.1174	0.1154	1.0	0.1188	0.1196	0.1170	0.1144
	2.2	0.1756	0.1892	0.1924	0.1896	1.2	0.3930	0.3904	0.3876	0.3976
	2.4	0.2560	0.2768	0.2776	0.2952	1.4	0.6786	0.6784	0.6762	0.6950
	2.6	0.3666	0.3660	0.3692	0.3994	1.6	0.8644	0.8710	0.8694	0.8740
	2.8	0.4298	0.4808	0.4596	0.5120	1.8	0.9540	0.9508	0.9570	0.9632
	3.0	0.5484	0.5516	0.5492	0.6032	2.0	0.9860	0.9836	0.9820	0.9916
100	2.0	0.0458	0.0486	0.0506	0.0504	1.0	0.0500	0.0498	0.0532	0.0560
	2.2	0.1024	0.1110	0.1112	0.1154	1.2	0.3536	0.3666	0.3648	0.3646
	2.4	0.1962	0.2160	0.1974	0.2258	1.4	0.7608	0.7596	0.7562	0.7792
	2.6	0.3146	0.3222	0.3160	0.3674	1.6	0.9480	0.9426	0.9446	0.9584
	2.8	0.4230	0.4424	0.4480	0.5032	1.8	0.9932	0.9922	0.9894	0.9936
	3.0	0.5670	0.5800	0.5692	0.6410	2.0	0.9988	0.9986	0.9990	0.9999

**Table 5:** Datasets X and Y corresponding survival times of Groups I and II.

Group I	159, 189, 191, 198, 200, 207, 220, 235, 245, 250, 256, 261, 265, 266, 280, 343, 356, 383, 403, 414, 428, 432, 317, 318, 399, 495, 525, 536, 549, 552, 554, 557, 558, 571, 586, 594, 596, 605, 612, 621, 628, 631, 636, 643, 647, 648, 649, 661, 663, 666, 670, 695, 697, 700, 705, 712, 713, 738, 748, 753, 40, 42, 51, 62, 163, 179, 206, 222, 228, 252, 249, 282, 324, 333, 341, 366, 385, 407, 420, 431, 441, 461, 462, 482, 517, 517, 524, 564, 567, 586, 619, 620, 621, 622, 647, 651, 686, 761, 763.
Group II	158, 192, 193, 194, 195, 202, 212, 215, 229, 230, 237, 240, 244, 247, 259, 300, 301, 321, 337, 415, 434, 444, 485, 496, 529, 537, 624, 707, 800, 430, 590, 606, 638, 655, 679, 691, 693, 696, 747, 752, 760, 778, 821, 986, 136, 246, 255, 376, 421, 565, 616, 617, 652, 655, 658, 660, 662, 675, 681, 734, 736, 737, 757, 769, 777, 800, 807, 825, 855, 857, 864, 868, 870, 870, 873, 882, 895, 910, 934, 942, 1015, 1019.

**Table 6:** Statistical Test Results for Real Data Set.

**Test**	$L_{0.5,N}$	$S_{N}$	$t_{N}$	${\hat{\Delta }}_{2,3,1.1}$
*p*-value	0.003300	0.000200	0.000556	0.001509

**Table 7:** Life test (hours) of two different snubber designs.

Old design	90, 100, 160, 346, 407, 456, 470, 494, 550, 570, 649, 733, 777, 836, 965, 983, 1008, 1164, 1474, 1550, 1576, 1620, 1643, 1705, 1835, 2043, 2113, 2214, 2422.
New design	23, 284, 371, 378, 498, 512, 574, 621, 846, 917, 1163, 1184, 1226, 1246, 1251, 1263, 1383, 1394, 1397, 1411, 1482, 1493, 1507, 1518, 1534, 1624, 1625, 1641, 1693, 1788.

**Table 8:** Statistical Test Results for the Second Real Data Set.

Test	$L_{0.5,N}$	$S_{N}$	$t_{N}$	${\hat{\Delta }}_{2,3,1.1}$
*p*-value	0.23524	0.20542	0.24778	0.32773

## Data Availability

The original contributions presented in this study are included in the article. Further inquiries can be directed to the corresponding author.

## Appendix A

R-code to find ξ ^^^_(N,α) by simulation xihat<-function(x,alpha,k,n){ x <- sort(x) g <- function(u) { (n-k+1)*(1-u)^k-(n-k)*(1-u)^(k+1) } g1 <- function(u) { k*(n-k+1)*(1-u)^(k-1)-(k+1)*(n-k)*(1-u)^(k) } J_alpha <- function(u) { (g1(u) / (alpha - 1)) * (1 - alpha * g(u)^(alpha - 1)) } sum<-0 for(i in 1:N){ sum<-sum+J_alpha(i/N)*x[i] } return(sum/N) } R-code to find Δ ^^^_(k,n,α) by simulation testD4 <- function (N,M,alpha,r,n){ x<-rexp(N) y<-rexp(M) N <- length(x) M <- length(y) deltanm <- f(y,alpha,r,n)-f(x,alpha,r,n) return(deltanm) } testDD4 <- function (N,M,b,alpha,r,n){ x<-rexp(N) y<-rexp(M,b) N <- length(x) M <- length(y) deltanm <- f(y,alpha,r,n)-f(x,alpha,r,n) return(deltanm) } N=M=25 alpha=2 k=2 n=3 q=0.05 b=0.5 z4 <- quantile(replicate(5000, testD4(N,M,alpha,r,n)), 1 - q) f4 <-function(b) mean(replicate(5000, testDD4(N,M,b,alpha,r,n) > z4), na.rm = TRUE)
